# Large-scale field application of a fingerstick blood test for *Mycobacterium leprae* infection: Monitoring population-wide effects of case finding and post-exposure prophylaxis on transmission in the Comoros and Madagascar

**DOI:** 10.1371/journal.pgph.0005270

**Published:** 2025-12-02

**Authors:** Louise Pierneef, Sofie M. Braet, Danielle de Jong, Stéphanie Ramboarina, Anouk van Hooij, Mohamed Wirdane Abdou, Nimer Ortuno-Gutierrez, Rian Snijders, Luca Maharavo, Silahi Halifa Grillone, Andriamira Randrianantoandro, Mala Rakoto-Andrianarivelo, Tahinamandranto Rasamoelina, Epco Hasker, Paul Corstjens, Bouke C. de Jong, Bertrand Cauchoix, Younoussa Assoumani, Annemieke Geluk

**Affiliations:** 1 Leiden University Center for Infectious Diseases, Leiden University Medical Center, Leiden, The Netherlands; 2 Institute of Tropical Medicine, Antwerp, Belgium; 3 Dept. Cell and Chemical Biology, Leiden University Medical Center, Leiden, The Netherlands; 4 Raoul Follereau, Antananarivo, Madagascar; 5 National Tuberculosis and Leprosy control Program, Moroni, Union of the Comoros; 6 Medical Technical Unit, Damien Foundation, Brussels, Belgium; 7 Centre d’Infectiologie Charles Mérieux, Antananarivo, Madagascar; 8 National Leprosy Program, Antananarivo, Madagascar; PLOS: Public Library of Science, UNITED STATES OF AMERICA

## Abstract

Since 2018, the WHO recommends provision of single-dose rifampicin post-exposure prophylaxis (SDR-PEP) to contacts of leprosy patients. In the Post-ExpOsure Prophylaxis for LEprosy (PEOPLE) trial that took place in the Comoros (Anjouan and Mohéli) and Madagascar (2019–2023), single double-dose rifampicin (SDDR)-PEP administration modalities were compared. Additionally, in 16/64 villages, anti-*M. leprae* phenolic glycolipid-I (anti-PGL-I) IgM levels were measured for three consecutive years, in newly diagnosed leprosy patients and more than 17,000 household- and neighbourhood contacts who had been screened for signs of leprosy. To study the impact of active case finding and SDDR-PEP on *M. leprae* infection rates in the population, fingerstick blood of contacts was sampled before, during and after the administration of SDDR-PEP. In this serosurvey, anti-PGL-I IgM levels were measured for the first time at such large scale by local staff using a field-friendly, quantitative lateral flow assay based on the UCP-platform (UCP-LFA). Out of 53 multibacillary patients with a bacterial index of 1 or higher, 92.5% (n = 49) tested seropositive for anti-*M. leprae* PGL-I IgM, confirming excellent performance of the test when executed by local staff. Before SDDR-PEP administration, seroprevalence among contacts in the different villages ranged from 21.9% to 27.4% across the three study sites. After two years of active case finding and SDDR-PEP, seroprevalence was significantly lower in the population in all three study sites (11.6% to 22.8%). Longitudinal serological monitoring in initially seropositive contacts showed significant reductions in anti-PGL-I IgM levels at an individual level. This reduction in seropositivity was observed irrespective of administration of SDDR-PEP. These serological data suggest that the implemented combination of active case finding with SDDR-PEP, led to a reduction in infection levels in the population in these study sites. This large-scale field serosurvey demonstrates the usefulness of the anti-PGL-I UCP-LFA to evaluate and monitor at a population level the effects of leprosy control interventions on *M. leprae* transmission.

## Introduction

Leprosy, caused by *Mycobacterium leprae* or *M. lepromatosis*, is a neglected tropical disease primarily affecting the skin and peripheral nerves [1,2]. The disease remains a significant public health issue in many parts of the world, particularly in parts of Asia and South-America [3], but also Africa [4]. Although in the WHO African Region, almost all Member States have achieved leprosy elimination (prevalence rate less than 1 per 10,000 population), pockets of hotspots within this region persist with more than 20,000 new cases reported every year [4].

Household contacts of leprosy patients - especially those with a high bacterial load - are most at risk of contracting the disease [5–7]. Early diagnosis and treatment are crucial for preventing the onset of disabilities and controlling transmission [3]. However, leprosy diagnosis remains clinical and therefore prone to miss asymptomatic individuals [8], underscoring the need for improved diagnostic tools.

Since 2018, the WHO has recommended provision of post-exposure prophylaxis (PEP) using single-dose rifampicin (SDR) to contacts of leprosy patients [9]. SDR-PEP should halt infection with *M. leprae* from progressing into clinical leprosy disease [10]. However, in previous studies using rifampicin as prophylaxis, contradictory results have been found: in Bangladesh, the COLEP trial (2002–2007) found a significant reduction in the risk of leprosy after SDR was given to contacts [11]. In contrast, a study conducted on five islands in the Flores Sea of Indonesia (2000–2003) showed no protective effect from two doses of rifampicin, three months apart, to household- and social contacts of leprosy patients [12]. However, this same study in Indonesia showed a four-fold decrease in leprosy incidence when PEP was offered as a blanket intervention across the entire island population.

The combination of active case finding and provision of single double-dose rifampicin (SDDR)-PEP to contacts of leprosy patients is aimed at reducing disease as well as breaking the chain of transmission [10,13]. The effectiveness of these interventions, however, requires monitoring through reliable tools that can detect changes in transmission and not only in the number of new cases. The PEOPLE trial, conducted on the Comoros and in Madagascar located in the Indian Ocean, was designed to explore SDDR-PEP administration modalities [14,15]. In the trial, 64 villages were randomly assigned to four arms and inhabitants were screened once a year for leprosy [14,15].

The current study describes the serological data collected using a quantitative, field-friendly test designed to detect present or past infection through measurement of *M. leprae*-specific anti-PGL-I IgM, which was included in one arm of the PEOPLE trial. The here assessed test utilizes luminescent, background-free, up-converting reporter particles (UCP) [16] and immunochromatography (i.e., the UCP-LF test platform) for accurate quantitation of anti-PGL-I IgM without operator bias [17]. Previously, it was demonstrated that the presence of anti-PGL-I IgM is highly specific for *M. leprae* infection, and that the level of anti-PGL-I IgM correlates strongly with the bacterial load present in an individual [18,19]. In this study, we applied the anti-PGL-I UCP-LFA for the first time at such large scale to assess the feasibility of measuring seroprevalence among leprosy patients and their contacts. Moreover, we aimed to evaluate the possibility of monitoring the impact of the implementation of active case finding and SDDR-PEP on *M. leprae*-specific antibody levels over time using our field-friendly test, providing insights into the effects of these interventions on *M. leprae* transmission.

## Materials and methods

### Study design and setting

The set-up of the PEOPLE trial in the Comoros and Madagascar has been described in extensive detail (https://clinicaltrials.gov/ct2/show/NCT03662022) [14,15]. The current study is focused on data gathered with the anti-PGL-I UCP-LFA and took place from January 11, 2019 to November 1, 2022. During this period, fingerstick blood (FSB) samples were collected from all consenting newly diagnosed leprosy cases on the Comoros (Anjouan and Mohéli; starting in 2019) and in Madagascar (Miandrivazo; starting in 2020) all newly diagnosed leprosy cases detected in the villages participating in the PEOPLE trial. Furthermore, FSB samples of leprosy contacts were collected in 16 villages that were assigned to arm 4 during the PEOPLE trial (Comoros: 9 on Anjouan; 3 on Mohéli; Madagascar: 4 on Miandrivazo). All samples were analysed using the anti-PGL-I UCP-LFA in local laboratories (Comoros: laboratory of the reference hospital Hombo in Mutsamudu on Anjouan; laboratory of the reference hospital in Fomboni on Mohéli; Madagascar: laboratory of the Centre d’Infectiologie Charles Mérieux in Antananarivo). From 2014-2019, the Comoros reported an annual incidence rate of 4.2 per 10,000 whereas on Madagascar 0.6 per 10,000 was reported [15,20]. Transmission in the areas remains high, as reflected by 36% of new cases being children (2018; Comoros), while nearly 20% of newly diagnosed cases in Madagascar have grade-2 disabilities (G2D) [14,20].

### Door-to-door screening

Annual screening rounds were conducted over a four-year period in each village where participants were recruited. Permanent residents of the study villages were invited for leprosy screening by national tuberculosis and leprosy programme (NTLP) teams in the Comoros, and national leprosy programme teams in Madagascar accompanied by community health workers during household visits. During household visits, all consenting household members present were screened for leprosy, and their history of previous leprosy diagnosis or treatment, tuberculosis (TB), and presence of a Bacillus Calmette–Guérin vaccine (BCG) scar were assessed. Individual eligibility criteria were based on WHO recommendations for SDR-PEP - i.e., healthy contacts aged 2 years and older, excluding possible patients with leprosy or tuberculosis, or both, in the absence of other contraindications.

### Leprosy patients

Newly identified patients during door-to-door screening or routine activities were treated according to NTLP guidelines [15]. Diagnosis of leprosy was clinical, based on the presence at least one of three cardinal signs: patch with loss of sensation, enlarged peripheral nerves and/or slit-skin smear (SSS) positive for acid fast bacilli. Patients were classified as PB (one to five lesions) or MB (more than five lesions) per WHO criteria [3]. For most MB patients, the bacterial index (BI) was determined using slit skin smears. Patients with disease detected after being screened during at least one prior round were classified as “incident cases” as they were already under contact-follow-up of the PEOPLE trial at time of their diagnosis.

### Contacts and SDDR-PEP

In the study villages, all eligible contacts were offered double (20 mg/kg) the regular dose (10 mg/kg) of SDR-PEP, based on an increased early bactericidal effect observed for *Mycobacterium tuberculosis* with this dose [21,22]. Paediatric dosages were adjusted according to body weight. Eligibility for SDDR-PEP was determined both on geospatial and individual criteria. All household contacts of newly diagnosed leprosy cases, as well as individuals living within 100m of a newly diagnosed case and testing seropositive for anti-PGL-I antibodies using the UCP-LFA were revisited. Those present and meeting individual eligibility criteria were offered SDDR-PEP. Individual eligibility criteria for SDDR-PEP included: healthy contacts aged two years and above, able and willing to provide informed consent. Exclusion criteria for SDDR-PEP included: signs of active leprosy, signs of active pulmonary TB (cough ≥ two weeks), and rifampicin intake within the last 24 months (to avoid any theoretical risk of inducing rifampicin resistance). SDDR-PEP was aimed to be distributed within one month following the completion of the annual screening round in each village, based on the cases detected during these screening rounds and anti-PGL-I test results.

### Informed consent procedures

Written informed consent was obtained for each individual participating in door-to-door screening. A separate written informed consent was collected for all leprosy patients prior to sampling. For those under 18 years old, written informed consent or parent/guardian approval was obtained, along with assent from minors aged 12 years or older.

### Fingerstick blood (FSB) collection

Prior to field activities, tubes with 980 μl high salt fingerstick (HSFS) buffer: 100mM Tris pH 8.0, 270mM NaCl, 1% (v/v) Triton X-100, and 1% (w/v) BSA were prepared. FSB was collected by local field staff using disposable 20 μl Minivette collection tubes (Heparin coated; Sarstedt) and added to the prepared buffer tubes on which the participants unique pseudonymized identification number was written. After sampling, FSB was transported at ambient temperature (in black plastic bags) to local laboratories where FSB samples were tested using UCP-LF strips. On the Comoros, during door-to-door screening entire village populations were sampled, whereas in Madagascar, only household contacts and individuals residing within 100m of a new case were sampled.

### Lateral flow strips

UCP-LF strips specific for anti-PGL-I IgM were produced in-house in four batches by Leiden University Medical Center as described previously [18,23]; total production size was 47,000. Synthesized disaccharide epitope (3,6-di-O-methyl-β-D-glucopyranosyl(1 → 4)2,3-di-O-methylrhamnopyranoside), similar to *M. leprae* specific PGL-I glycolipid, coupled to human serum albumin (synthetic PGL-I; designated ND-O-HSA) was obtained through the Biodefense and Emerging Infections Research Resources Repository (http://www.beiresources.org/TBVTRMResearchMaterials/tabid/1431/Default.aspx).

The Test (T) line on the LF strip (nitrocellulose membrane; Sartorius UniSart CN95) comprised 100 ng of synthetic PGL-I. The flow-control (FC) line comprised 100 ng rabbit anti-goat IgG (G4018; Sigma-Aldrich, Inc., St. Louis, MO, USA). Goat IgG specific for anti-human IgM (I0759; Sigma-Aldrich, Inc., St. Louis, MO, USA) was conjugated to polyacrylic acid functionalized UCPs [200 nm, NaYF4:Yb3 + , Er 3 + ; Intelligent Material Solutions Inc. (IMS); Princeton, NJ, USA MS] according to previously described protocols at a concentration of 50 μg antibody per mg UCP [24]. UCP stock solutions were kept at 4°C until use. To dry the UCPs on the glass fiber conjugate-release pad, the material was diluted in a buffer containing 100 mmol/L Tris pH 8.0, 270 mmol/L NaCl, 10% (w/v) sucrose, 1% (w/v) BSA, 0.5% Tween-20, and striped at a density of 100 ng/mm. Components were mounted on plastic backing cards which were cut into LF strips of 4 mm width by 5 cm length.

### UCP-LFA

In the local laboratories, each UCP-LF strip was labeled with the same written unique barcode (as on the tube) specific to a participant. 50 µl of the in the field stabilized, diluted, and lysed (with HSFS buffer) FSB sample was added to a 96-wells plate and LF was initiated by placing the UCP-LF strip into the well. Immunochromatography was allowed to continue until strips were dry (overnight). UCP-LF strips were scanned with a UCP dedicated benchtop reader (Madagascar; UPCON; Labrox Oy Turku, Finland) and a standard portable ESEQuant *LFR* adapted for UCP (Comoros; DIALUNOX, Stockach, Germany). Results were calculated as the ratio (R) value between Test (T) and Flow Control (FC) signal based on relative fluorescence units (RFUs) measured at the respective lines. A quality control (QC) protocol was designed to monitor test reproducibility over time using sera of clinically diagnosed leprosy patients selected based on their anti-PGL-I IgM levels in standard anti-PGL-I IgM ELISAs [18,25]: a highly seropositive, a seropositive with an OD around the cut-off for seropositivity in anti-PGL-I IgM ELISAs (QC reference) and a seronegative serum sample (from a healthy Dutch blood bank donor without travel history to leprosy endemic areas). As described above, each site used their own UCP-reader which were from different suppliers. Sites were provided with UCP-LF strips, comprising different production lots, with regular shipments. To allow direct comparison of all test results from different batches and readers, results were normalized and presented as anti-PGL-I ‘units’ using the QC reference serum sample. First, cut-offs for different strip batches were determined as the median R-value of 20 measurements of the QC reference serum sample minus the standard deviation for that respective UCP-LF batch. Units were then determined by dividing individual R-values by the R-value of the cut-off for that respective UCP-LF strip batch, like normalization methods used in anti-PGL-I ELISAs [26–29]. Units ≥ 1 are considered seropositive.

### Ethics

This study was performed according to the Helsinki Declaration (7^th^ revision, 64^th^ Meeting, 2013, Fortaleza). Ethical approval of the trial protocol was obtained through the ‘Comité d’Éthique de la Recherche Biomédicale’ (CERBM) in Madagascar and the ‘Comité National d’Éthique pour les Sciences de la Vie et de la Santé’ (CNESS) in the Comoros. Approval was also obtained from the Institutional Review Board (IRB) of ITM and the Ethics Committee (EC) of the University of Antwerp Hospital. Prior to the start, this trial was included in the Clinicaltrials.gov public registry (NCT03662022, on September 7^th^ 2018, https://clinicaltrials.gov/ct2/show/NCT03662022).

### Statistical analysis

GraphPad Prism version 9.0.1 for Windows (GraphPad Software, San Diego, CA, USA) and IBM SPSS Statistics for Windows version 29 (IBM Corp, Armonk, NY, USA) were used to perform statistical analysis. Analyses of quantitative anti-PGL-I units were performed using the Mann-Whitney U test for comparisons between two groups and the Kruskal-Wallis test with Dunn’s correction for multiple testing for comparisons between three groups. Paired analyses of anti-PGL-I units were conducted using the Wilcoxon signed-rank test for two groups and the Friedman test with Dunn’s correction for multiple testing for three groups. To evaluate statistical significance in seroprevalence between study years in cross-sectional analyses, the Chi-squared test was applied. For paired comparisons of seroprevalence between study years, the McNemar’s test was used. The analysis on contact data included data from incident cases gathered prior to their leprosy diagnosis, but excluded data collected after their diagnosis.

## Results

To determine seroprevalence and quantitatively monitor anti-PGL-I IgM levels over time in the leprosy endemic areas where active case finding and SDDR-PEP administration were implemented during the PEOPLE trial, FSB samples from index cases (n = 702) as well as their household and neighbourhood contacts (n > 17,000) were obtained during field screenings.

### Patients

**Fig 1:**
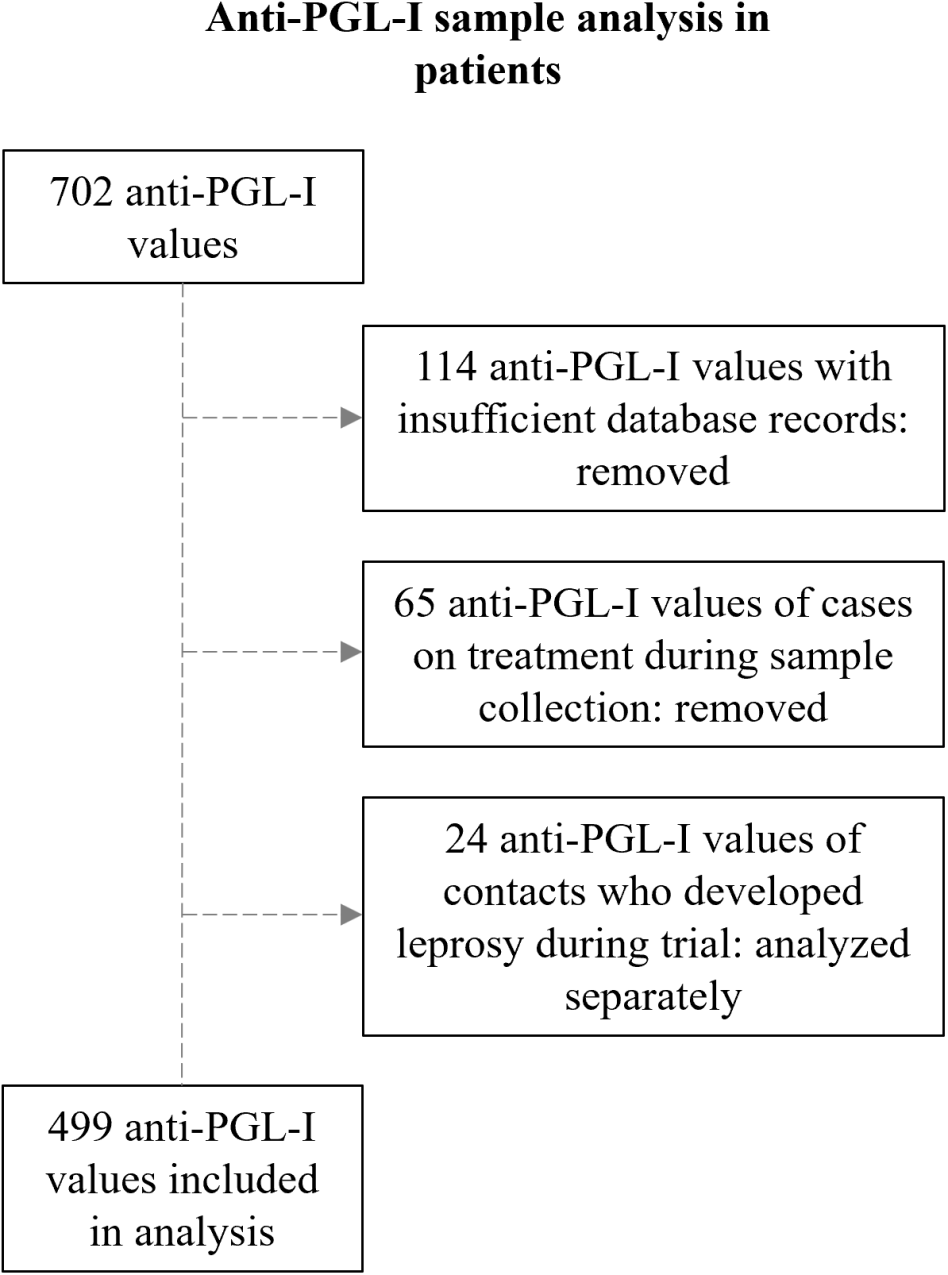
Schematic representation of data preprocessing of FSB samples included in the analysis of anti-PGL-I values of leprosy patients. Insufficient database records included missing date of diagnosis or sampling. PGL-I: phenolic glycolipid-I.

To first assess the field performance of the anti-PGL-I UCP-LFA in leprosy patients by local staff on the Comoros, leprosy patients detected during door-to-door screening or routine leprosy case detection were tested for anti-PGL-I IgM at field sites using the anti-PGL-I UCP-LFA. Out of the 1,369 patients diagnosed between 2019 and 2022 (Comoros: 1,241 on Anjouan, 128 on Mohéli), 702 (51%) anti-PGL-I values were available. Of these 702 anti-PGL-I values, 114 were excluded because they were missing key information such as date of diagnosis or sampling. Additionally, 65 anti-PGL-I values were removed from the analysis as the patients had already started multi-drug therapy (MDT) at the time of first sampling (Fig 1). Anti-PGL-I values of individuals who were initially included as contacts but developed leprosy during the study (incident cases; n = 24) were also excluded from this evaluation as they were analysed separately. Of the 499 leprosy patients remaining, 162 (32.5%) were clinically diagnosed with MB and 337 (67.5%) with PB leprosy (Table 1).

**Table 1:** Overview of leprosy patients from the Comoros tested for anti-PGL-I IgM at diagnosis between 2019 and 2022. Leprosy type and bacterial index are indicated if available.

Leprosy patients	*n*	%
**MB**	**162**	**32.5**
BI		
0	91	18.2
1	17	3.4
2	13	2.6
3	8	1.6
4	7	1.4
5	4	0.8
6	4	0.8
nd	18	3.6
**PB**	**337**	**67.5**
BI		
nd	337	67.5
**Total**	**499**	**100**

BI: bacterial index; MB: multibacillary; PB: paucibacillary; nd: not determined.

**Fig 2:**
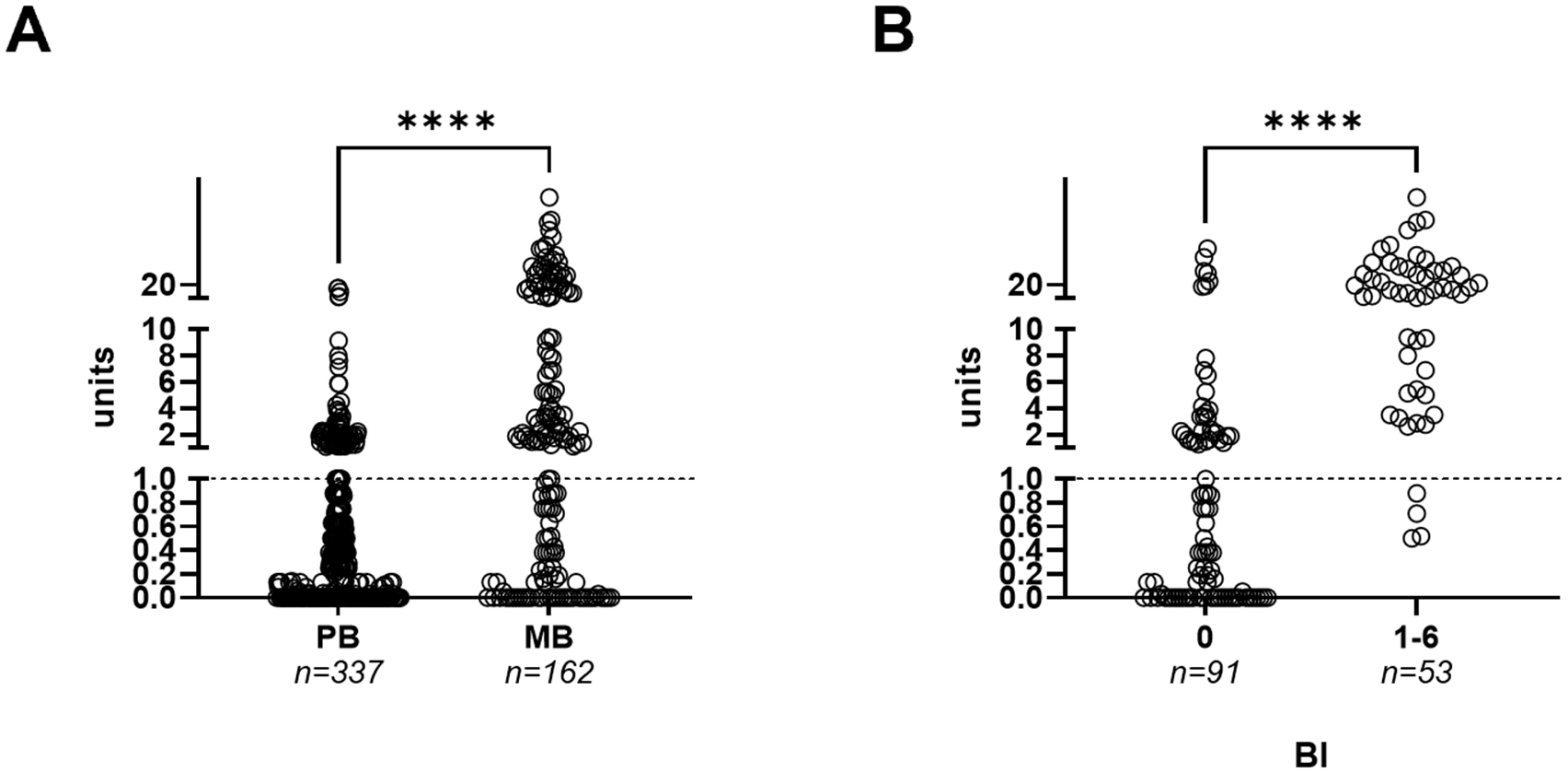
Anti-PGL-I values (shown in units) in FSB of PB and MB leprosy patients. A: Anti-PGL-I IgM (*y*-axis) units among PB (n = 337) and MB (n = 162) leprosy patients (*x*-axis). B: Anti-PGL-I IgM (*y*-axis) units among leprosy patients stratified per bacterial index (BI) group (0 and 1-6; *x*-axis). Open circles represent anti-PGL-I units for individual samples. Units above the dotted line (units ≥ 1) are considered seropositive. The Mann-Whitney U test was performed to test for statistical significance (****P ≤ 0.0001). BI: bacterial index; FSB: fingerstick blood; IgM: immunoglobulin M; MB: multibacillary; PB: paucibacillary; PGL-I: phenolic glycolipid-I.

Anti-PGL-I units among patients ranged from 0 to 86.29. 174 out of 499 patients (34.9%) tested seropositive for anti-PGL-I IgM; 80 out of 337 PB patients (23.7%) and 94 out of 162 MB patients (58.0%) (Fig 2). Patients with a BI of 1 and higher had significantly increased anti-PGL-I units compared to those with BI 0 (Mann-Whitney: *P* ≤ 0.0001). Importantly, among MB patients testing anti-PGL-I seronegative for which their BI was available (n = 61), 93.4% (n = 57) had a BI of 0. When only MB patients with a BI of 1 or higher were considered, 49 out of 53 (92.5%) tested seropositive for anti-PGL-I IgM, showing a strong association between anti-PGL-I antibodies and bacterial load. These results confirm the previously reported high sensitivity of the anti-PGL-I UCP-LFA in detecting MB leprosy cases [17].

### Contacts

**Fig 3:**
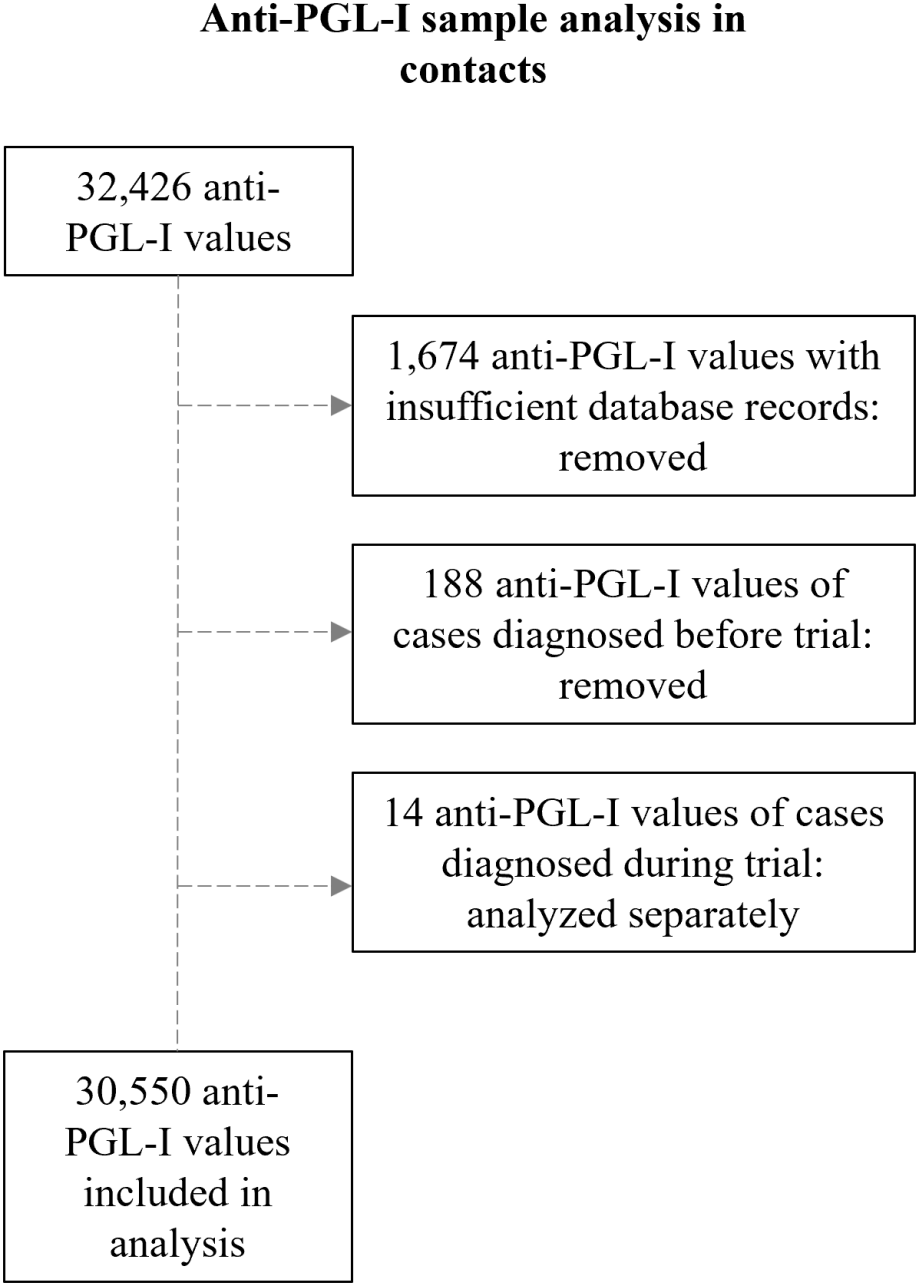
Schematic representation of data preprocessing of FSB samples included for analyses of anti-PGL-I values of contacts. Insufficient database records included incomplete/errors in barcodes or missing date of sampling. PGL-I: phenolic glycolipid-I.

To evaluate the feasibility of a large-scale field serosurvey among contacts of leprosy patients in the Comoros and Madagascar using the anti-PGL-I UCP-LFA, a total of 32,426 tests were conducted between 2019 and 2022 (Fig 3). A total of 1,674 anti-PGL-I values were excluded due to incomplete/errors in barcodes or missing date of sampling. Additionally, 188 anti-PGL-I values derived from individuals who were identified as (former) patients after their initial inclusion (as contacts), of which no data from prior to - or at timepoint of diagnosis were available, were removed from the dataset. Furthermore, 14 anti-PGL-I values (from after diagnosis) corresponding to contacts who were diagnosed with leprosy during the study period were moved to a separate analysis, as illustrated in S3 Fig. Consequently, 30,550 anti-PGL-I values remained for the analysis.

A total of 12,496 individuals were included as contacts and tested for anti-PGL-I IgM in year 1: 9,642 from Anjouan, 2,300 from Mohéli and 554 from Madagascar (Table 2).

**Table 2:** Overview of contacts included and tested for anti-PGL-I IgM per study site and year on the Comoros and in Madagascar between 2019 and 2022.

Contacts		Year 1	Year 2	Year 3
Anjouan	Total	9642	5547	7191
	Sex			
	Male	4,201 (43.6%)	2,192 (39.5%)	2,893 (40.2%)
	Female	5,441 (56.4%)	3,355 (60.5%)	4,298 (59.8%)
	Median age (range in years)	17 (2-100)	15 (2-100)	15 (2-100)
Mohéli	Total	2300	1984	2071
	Sex			
	Male	926 (40.3%)	779 (39.3%)	787 (38.0%)
	Female	1,374 (59.7%)	1,205 (60.7%)	1,284 (62.0%)
	Median age (range in years)	20 (2-93)	19 (2-92)	18 (2-91)
Madagascar	Total	554	571	690
	Sex			
	Male	252 (45.5%)	278 (48.7%)	330 (47.8%)
	Female	302 (54.5%)	293 (51.3%)	360 (52.2%)
	Median age (range)	18 (2-92)	16 (2-90)	17 (2-90)
All sites	Total	12,496	8,102	9,952

**Fig 4:**
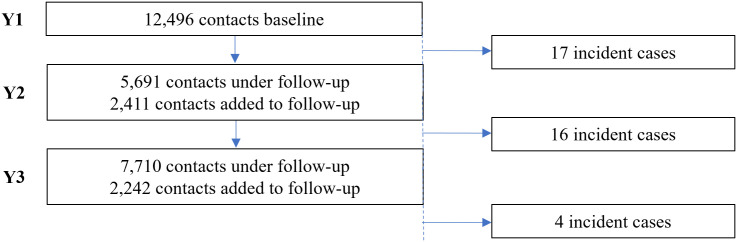
Flow diagram showing follow-up of contacts per study year. Patients with leprosy diagnosed after the first screening round were classified as “incident cases” if already under contact-follow-up of the PEOPLE trial at time of their diagnosis. A total of 37 incident cases were diagnosed among individuals included for anti-PGL-I testing (PB n = 25; MB n = 12; S1 Table).

In year 2 of the study, 8,102 contacts were tested for anti-PGL-I IgM, of whom 5,691 (70.2%) already had been tested in year 1 (Fig 4). In year 3 of the study, 9,952 contacts were tested and 7,710 (77.5%) of those had already been tested in year 1 and/or year 2.

37 contacts of whom anti-PGL-I data were available were diagnosed with leprosy during the study period; 35 from Anjouan and 2 from Mohéli (Fig 4). These individuals were first included as contacts (showing no signs of leprosy) and at some point during the study period – after baseline inclusion – diagnosed with leprosy (incident cases; S1 Table). Seventeen contacts were diagnosed with leprosy between sample collections in year 1 and 2, whereas between sample collections in year 2 and 3, 16 contacts were diagnosed with leprosy. Four more contacts developed leprosy after the 3^rd^ year of sample collection (up to November 2022). In total, 12 of these 37 (32.4%) incident cases were classified as MB leprosy and 25 (67.6%) as PB leprosy.

**Fig 5:**
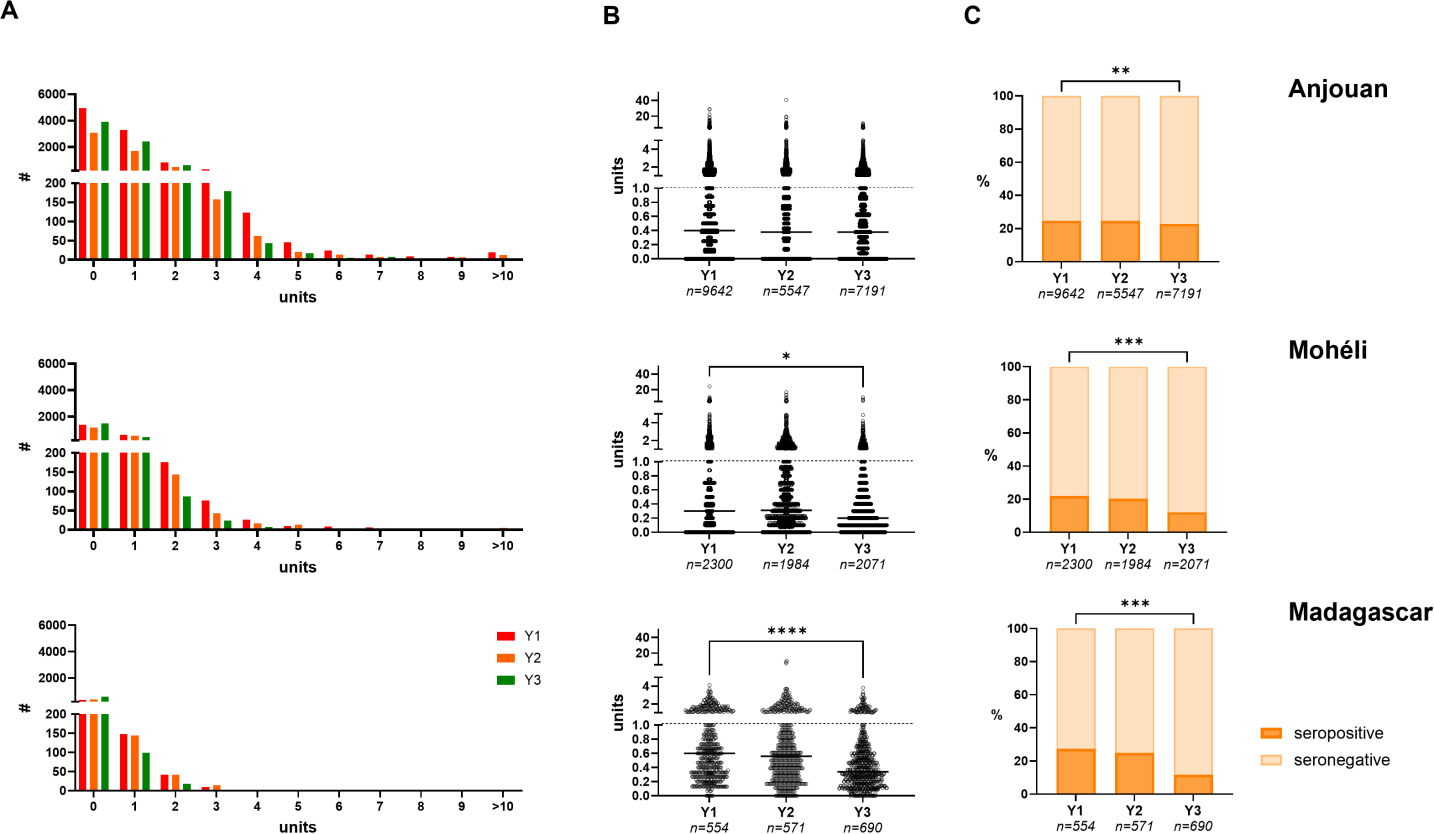
Anti-PGL-I values (shown in units) in FSB among contacts on Anjouan, Mohéli and Madagascar. A: Distribution of anti-PGL-I IgM units among contacts on Anjouan (upper panel), Mohéli (middle panel) and Madagascar (lower panel). The number (#) of tests (*y*-axis) per unit range (0-0.99; 1-1.99; etc.) are indicated on the *x*-axis. B: Anti-PGL-I IgM (*y*-axis) units of contacts on Anjouan, Mohéli and Madagascar per study year (*x*-axis). Open circles represent anti-PGL-I units for individual samples. Units above dotted line (units ≥ 1) are considered seropositive. Kruskal-Wallis tests were performed to test for statistical significance between anti-PGL-I units at the beginning and end of the study (*P ≤ 0.05, **P ≤ 0.01, ***P ≤ 0.001, ****P ≤ 0.0001). C: Percentages (%) of contacts testing seropositive (*y*-axis) for anti-PGL-I IgM depicted per study year (*x*-axis). Chi-squared tests were performed to test for statistical significance between percentages at the beginning and end of the study (*P ≤ 0.05, **P ≤ 0.01, ***P ≤ 0.001). FSB: fingerstick blood; IgM: immunoglobulin M; PGL-I: phenolic glycolipid-I; Y1: year 1; Y2: year 2; Y3: year 3.

Anti-PGL-I units among contacts ranged from 0 to 40.86. Median anti-PGL-I units were 0.38 (IQR 0-0.90) and 0.30 (IQR 0.10-0.70) for Anjouan and Mohéli respectively, where the whole village was tested, and 0.47 (IQR 0.23-0.86) for Madagascar where only contacts within a 100m radius of new cases were tested. All three distributions were strongly skewed to the right. In addition, the majority of individuals tested seronegative (Fig 5A). Median anti-PGL-I units were highest among individuals aged 11–40 (S1 Fig), in line with previous studies [30,31].

To study the effect of the activities in the PEOPLE trial on *M. leprae* infection in the study population over time, analysis between anti-PGL-I IgM levels at the beginning (year 1) and the end (year 3) of the study was performed. On Anjouan, no significant difference in median antibody levels between year 1 and 3 was observed (Fig 5B; Kruskal-Wallis: *P* > 0.999), whereas seroprevalence for anti-PGL-I IgM significantly decreased from 24.7% in year 1 to 22.8% in year 3 (Fig 5C; Chi-squared: *P* < 0.01). On Mohéli, seroprevalence declined from 21.9% in year 1 to 12.0% in year 3 (Chi-squared: *P* < 0.001) and median antibody levels also showed a significantly decreasing trend over the years (Kruskal-Wallis: *P* < 0.05). In Madagascar, anti-PGL-I seroprevalence decreased from 27.4% (year 1) to 11.6% (year 3; Chi-squared: *P* < 0.001), and median antibodies were significantly lower (Kruskal-Wallis: *P* < 0.0001). Overall, the implemented interventions (including treatment of index cases) appeared to have a reducing impact on the population’s infection levels.

### Children

To assess recent *M. leprae* transmission in the community, a sub-analysis on anti-PGL-I data from children (age 2–10 years) was conducted. Seroprevalence in young children reflects recent *M*. *leprae* infection and may thus be used to monitor transmission in an area [32–34]. Although median antibody levels significantly increased (Kruskal-Wallis: *P* < 0.01; S2A Fig) over the years in children up to ten years old on Anjouan (the island reporting the highest leprosy incidence in the preceding years), seroprevalence in children remained around 20.0% (Chi-squared: *P* = 0.492; S2B Fig). In contrast, median anti-PGL-I units in children on Mohéli and Madagascar were significantly decreased in year 3 compared to year 1 and/or 2 (Kruskal-Wallis: *P* < 0.0001) and seroprevalence decreased from 25.5% to 13.7% and 22.7% to 9.4% (Chi-squared: both *P* < 0.001), respectively.

### Incident leprosy cases

Anti-PGL-I levels before and at diagnosis of leprosy were compared in more detail: six out of 12 (50.0%) incident MB cases tested seropositive for anti-PGL-I IgM at some point during the study (S3 Fig); three incident cases who initially tested seronegative, were seropositive for anti-PGL-I IgM at the time of diagnosis. Another three individuals already tested seropositive before diagnosis. Out of the six MB patients testing seronegative, four (66.7%) had a BI of 0 and for the other two (33.3%) the BI was not available.

For incident PB cases, 20 out of 25 (80.0%) tested seronegative for anti-PGL-I IgM throughout the study, whereas five (20.0%) tested seropositive at least once. Although not significant (Mann-Whitney U: *P* = 0.08), higher units of anti-PGL-I were found for incident MB patients (n = 7) compared to incident PB patients (n = 14; S4 Fig). These results again confirm good performance of the test among BI-positive individuals.

Five out of 37 (13.5%) incident cases had received SDDR-PEP prior to leprosy diagnosis, of whom four tested seropositive (S3 Fig). Among them, three (60.0%) were diagnosed with MB and two (40.0%) with PB leprosy.

### Longitudinal anti-PGL-I IgM and SDDR-PEP among contacts

**Fig 6:**
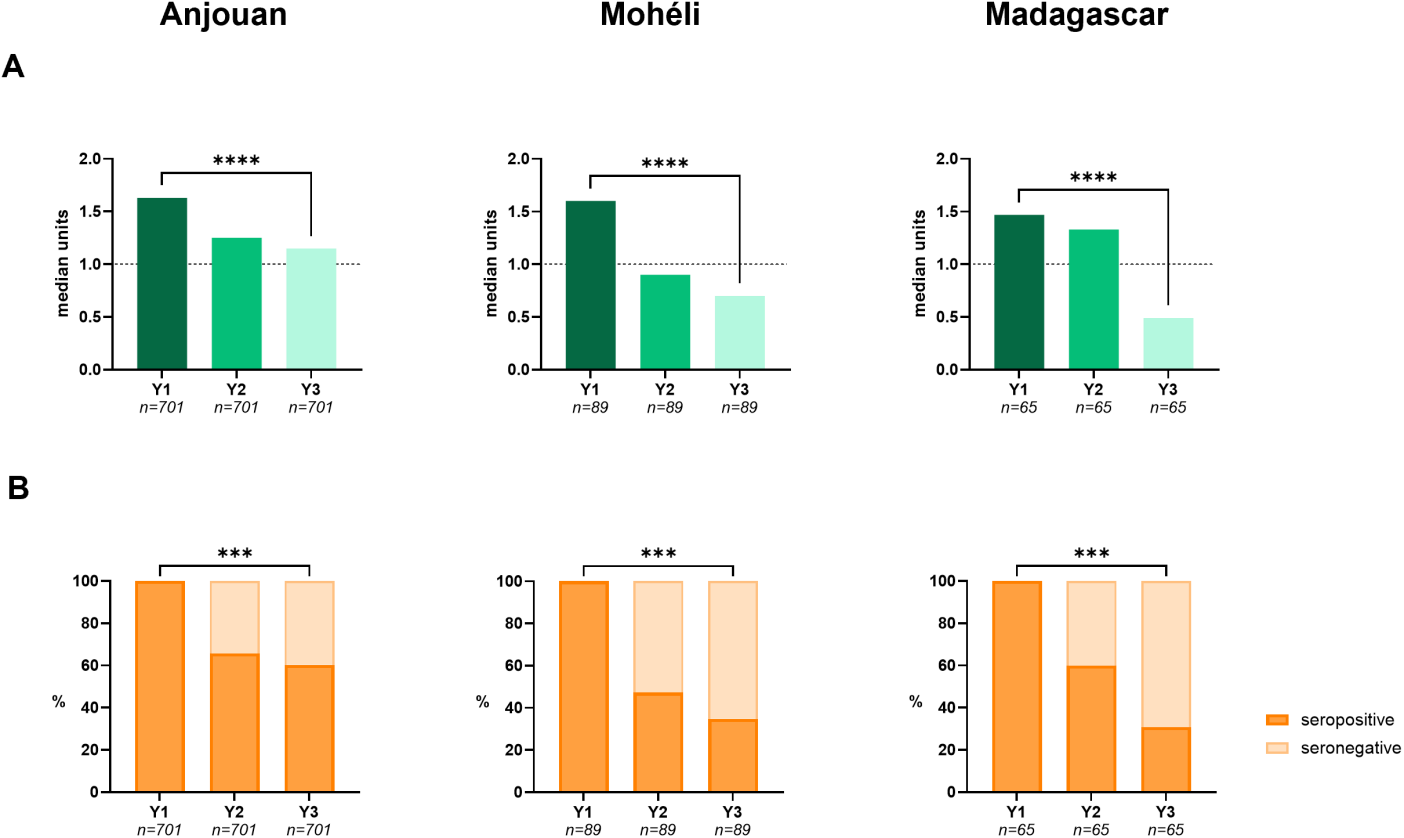
Longitudinal anti-PGL-I values (shown in units) in FSB of contacts on Anjouan, Mohéli and Madagascar testing seropositive in year 1. A: Paired median anti-PGL-I IgM units of seropositive contacts (Y1; *y*-axis) on Anjouan (left panel), Mohéli (middle panel) and Madagascar (right panel) are depicted per study year (*x*-axis). Anti-PGL-I units above dotted line (units ≥ 1) are considered seropositive. Friedman tests were performed to assess statistical significance between anti-PGL-I units at the beginning and end of the study (****P ≤ 0.0001). B: Paired percentages (%) of contacts testing seropositive (*y*-axis) for anti-PGL-I IgM depicted per study year (*x*-axis). McNemar tests were performed to assess statistical significance between percentages at the beginning and end of the study (***P ≤ 0.001). FSB: fingerstick blood; IgM: immunoglobulin M; PGL-I: phenolic glycolipid-I; Y1: year 1; Y2: year 2; Y3: year 3.

When analysing anti-PGL-I data longitudinally to study the effects of the implemented interventions on an individual level (irrespective of SDDR-PEP intake), contacts testing seropositive for anti-PGL-I IgM in year 1, showed significantly decreased antibody levels in year 3 (Friedman: *P* < 0.0001) and seroprevalence (McNemar: *P* < 0.001) (Figs 6 and S5) in all three study sites. While on Mohéli and Madagascar median anti-PGL-I units dropped below 1 (anti-PGL-I seronegative) in year 3, the median unit on Anjouan remained above 1.

As the prior analysis did not include individual SDDR-PEP intake, we next performed a paired analysis stratified by SDDR-PEP recipients and non-recipients. This analysis showed significant reductions in paired anti-PGL-I units in year 3 when compared to year 1 among seropositive contacts who received SDDR-PEP (S6A Fig; Wilcoxon: Anjouan and Madagascar *P* < 0.0001, Mohéli *P* ≤ 0.01). However, similar results were found among contacts who did not receive SDDR/PEP (Wilcoxon: Anjouan and Mohéli *P* < 0.0001, Madagascar *P* < 0.01). Amongst contacts seropositive in year 1, seroprevalence declined (from 100%) to 25.4% on Madagascar, 61.1% on Mohéli, and 61.5% on Anjouan in year 3 among PEP-recipients (McNemar: Anjouan and Madagascar *P* < 0.001, Mohéli *P* < 0.05). Among non-SDDR-PEP recipients, seroprevalence declined to 35.0%, 32.6%, and 62.6% on Madagascar, Mohéli, and Anjouan, respectively (McNemar: *P* < 0.001; S6B Fig).

## Discussion

Despite the availability of MDT, around 200,000 new persons are affected by leprosy each year [35], highlighting the need for innovations in leprosy control efforts [36]. Given that household contacts of untreated leprosy patients are at the greatest risk of infection [37], and that previous studies have demonstrated the effectiveness and feasibility of SDR-PEP, the WHO recommends the screening of close contacts of new leprosy patients combined with administration of SDR-PEP to prevent disease progression in potentially infected individuals [9].

This study describes the largest field application of the anti-PGL-I UCP-LFA to date (n = 33,128 tests), using humoral immunity in the population (anti-*M. leprae*-specific seroprevalence), to assess the impact of PEP implementation including repeated door-to-door screening on population infection levels, rather than merely evaluating the number of newly detected cases many years after PEP. While the primary objective of the PEOPLE trial was to explore the effects of various SDDR-PEP administration methods among contacts of leprosy patients across the Comoros and Madagascar, the focus of the present study was on the utility of the anti-PGL-I UCP-LFA in two use cases within this context. Our findings underscore the significant value of this quantitative field-friendly test in (1) diagnosing (MB) leprosy and (2) monitoring infection trends in populations undergoing leprosy control interventions.

The anti-PGL-I UCP-LFA exhibited excellent performance in detecting leprosy patients with a positive BI, with 92.5% (49/53) testing seropositive. As was also shown previously [17,18], a higher bacterial load was associated with higher anti-PGL-I units. Additionally, for 37 contacts who developed leprosy during the PEOPLE trial (incident cases), all four incident MB cases with a positive BI, tested seropositive at the time of diagnosis. These findings are consistent with previous studies and support the use of anti-PGL-I UCP-LFA, alongside clinical evaluation, as an adjunctive diagnostic tool for MB, offering a less invasive alternative to slit-skin smears or biopsies through FSB sampling [17–19,38]. Also in line with previous reports, the anti-PGL-I UCP-LFA demonstrated lower sensitivity in PB patients, of whom 23.7% (80/337) tested seropositive. This result reflects the challenge of detecting *M. leprae* infection in individuals with a negative or low BI using antibody-based tests, as these patients primarily mount a cellular immune response, and antibody levels - while sometimes detectable - are generally lower due to limited bacterial load and more efficient immune control [39]. Therefore, there is a need for complementary biomarkers for improved detection in this patient group [24,40,41].

Despite the fact that six out of 37 contacts (16.2%; n = 3 MB; n = 3 PB) who developed leprosy during the trial already tested seropositive (anti-PGL-I units 1 – 5) approximately one year (or more) before diagnosis, previous reports have shown that anti-PGL-I seropositivity is not predictive for leprosy disease [42–44]. In line with this, we found that 83.8% (n = 31) of incident cases tested anti-PGL-I seronegative one or two years prior to their diagnosis. However, as a result of a serosurvey conducted in India, a nine-year-old child who tested seropositive for anti-PGL-I IgM during a door-to-door serosurvey in Bihar, was subsequently diagnosed with PB leprosy during follow-up one year after the serosurvey. The opportunity for early detection of leprosy, including PB, through follow-up of seropositive individuals, underscores the additional benefit of serosurveys [45].

At the start of the study (year 1, before SDDR-PEP implementation), seroprevalence among contacts ranged from 21.9% to 27.4% across the three study sites. By year 3, seroprevalence on Anjouan was not very different from year 1 (8% reduction), while in Madagascar and Mohéli substantial reductions were observed (45% and 58%, respectively), suggesting a beneficial impact of active case finding and SDDR-PEP on infection rates. Despite decreased seroprevalence on Anjouan, no significant drop in anti-PGL-I levels was observed, possibly reflecting differences in transmission dynamics or intervention efficacy, potentially influenced by population density and leprosy incidence, both notably lower in Madagascar and Mohéli in comparison to Anjouan [19,46].

Still, in all study sites, paired analysis of seropositive individuals showed reduced anti-PGL-I levels over time, regardless of SDDR-PEP intake. Similarly, a study in South Sumatra, Indonesia, reported reduced anti-PGL-I IgM among seropositive contacts of leprosy patients two years after SDR-PEP [47]. However, our data regarding individual anti-PGL-I levels before and after PEP, suggest that the combination of active case finding followed by treatment of new cases and PEP - rather than PEP alone - play a crucial role in reducing *M. leprae* transmission and that this could successfully be measured using our anti-PGL-I UCP-LFA. In the PEOPLE trial, household contacts and anyone living within 100m of an index case and who tested seropositive for anti-PGL-I received PEP. Including community controls not receiving PEP in future studies could be informative to assess only the effects of PEP on anti-PGL-I IgM levels among contacts.

Notably, five individuals who received SDDR-PEP before diagnosis still progressed to clinical disease. Incorporating additional immunological markers beyond anti-PGL-I may enhance evaluation of prophylactic interventions, as is currently being investigated in the INDIGO#2 trial in Bangladesh (NCT06222372) [48].

With numerous initiatives being implemented to combat leprosy, including the administration of SDR-PEP [49–51], it is important to investigate the (long-term) effects of such prophylactic measures on the transmission of *M. leprae* and globally compare the impact of PEP across areas with different levels of endemicity. As infection in young children per definition reflects recent transmission in a population [32–34], testing should be focused on young children to assess seroprevalence within a community prior to and following the implementation of interventions. An analysis of the data for children alone showed that seroprevalence remained stable on Anjouan, whereas it significantly decreased in the other two study sites after intervention implementation, again indicating varied effects on recent transmission as discussed earlier. While some countries are progressing towards or have already successfully achieved the elimination of leprosy as determined by the number of new child cases [52,53], it remains essential to maintain robust surveillance systems [54]. In this context, the introduction of a serological rapid test that allows large-scale field-testing could represent a straightforward, yet effective approach to monitor transmission of *M. leprae*.

A key requirement for serosurveillance in leprosy endemic populations is the implementation of a standardized, quantitative assay that is user-friendly, allowing large-scale application in low resource field settings. When the PEOPLE trial began in 2019, a pre-final version of the current anti-PGL-I UCP-LFA format was available, which consisted of LF strips and vertical flow. In 2024, the LUMC immunodiagnostic research group introduced the PGL-I QURapid, which, besides having the advances of the UCP-LFA format also includes a biodegradable cassette [17]. Unlike the methodology used in this study (2019–2023), this new version including the biodegradable cassette, will eliminate the need for a 96-well plate, allowing the diluted FSB sample to be directly applied to the test cassette, making it significantly easier for field staff to use in future studies. Moreover, pre-printed barcode stickers can be applied to the cassette, reducing the risks occurring during writing of participant IDs.

## Conclusions

Our findings confirm the high sensitivity of the anti-PGL-I UCP-LFA in detecting BI-positive individuals. Moreover, the data of the serological analysis of the effect of SDDR-PEP in the Comoros and Madagascar show that concurrent with active case finding and PEP implementation, anti-PGL-I seroprevalence in the population, representing the number of individuals with past or present infection, significantly decreased in all three study sites. The seroprevalence decreased in both contacts who did and did not receive SDDR-PEP, albeit not randomized, suggesting that active case finding, with treatment initiation rapidly decreasing infectiousness, is probably at least as important in reducing transmission, as is PEP. Simultaneously, *M. leprae*-specific antibody levels, representing the extent of infection, significantly decreased over the years in the populations of Mohéli and Madagascar. Thus, this study highlights the suitability of the low-invasive, field-friendly anti-PGL-I UCP-LFA, for large-scale implementation in leprosy control programs [17]. By enabling the detection of past or present infection with *M. leprae* in a quantitative fashion this test can play a vital role in monitoring the effectiveness on transmission of control interventions, thus providing an essential tool for the global effort to reduce the burden of leprosy.

## Supporting information

S1 Table Overview of contacts tested for anti-PGL-I prior to leprosy diagnosis. *No incident cases in Madagascar were tested for anti-PGL-I IgM. MB: multibacillary; PB: paucibacillary.
(DOCX)

S1 Fig Anti-PGL-I values (shown in units) in FSB samples of contacts stratified by age. Open circles represent anti-PGL-I IgM units of individual samples. Units above dotted line (units ≥ 1) are considered seropositive. Kruskal-Wallis tests with Dunn’s correction for multiple testing were performed to assess statistical significance between anti-PGL-I units per age group.
(TIF)

S2 Fig Anti-PGL-I values (shown in units) in FSB samples of child contacts on Anjouan, Mohéli and Madagascar. A: Anti-PGL-I IgM (*y*-axis) units of children (2–10 years of age) who are contacts of leprosy patients on Anjouan (left panel), Mohéli (middle panel) and Madagascar (right panel) per study year (*x*-axis). Open circles represent anti-PGL-I units of individual samples. Units above dotted line (units ≥ 1) are considered seropositive. Kruskal-Wallis tests were performed to assess statistical significance between anti-PGL-I units at the beginning and end of the study (**P ≤ 0.01, ****P ≤ 0.0001). B: Percentages (%) of child contacts (2–10 years of age) testing seropositive for anti-PGL-I IgM (*y*-axis) depicted per study site and year (*x*-axis). Chi-squared tests were performed to assess statistical significance between percentages at the beginning and end of the study (***P ≤ 0.001). FSB: fingerstick blood; IgM: immunoglobulin M; PGL-I: phenolic glycolipid-I; Y1: year 1; Y2: year 2; Y3: year 3.
(TIF)

S3 FigLongitudinal monitoring of anti-PGL-I values (shown in units) in FSB of incident leprosy cases.Heat maps showing anti-PGL-I IgM units of MB (left panel; n = 12) and PB (right panel; n = 25) leprosy cases. Anti-PGL-I units ≥ 1 are considered seropositive. ‘P’ indicates timepoint of receiving SDDR-PEP. Triangles indicate timepoint of diagnosis. ‘
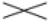
’ indicates that no FSB sample was taken in that year. Four contacts were diagnosed with leprosy after the third year of the study. Anti-PGL-I units 0-0.99: light orange; units 1-4.99: orange; units 5-9.99: dark orange; units ≥ 10: red. BI: bacterial index; FSB: fingerstick blood; MB: multibacillary; na: not available; PB: paucibacillary; PGL-I: phenolic glycolipid-I; SDDR-PEP: single double-dose rifampicin post exposure prophylaxis; Y1: year 1; Y2: year 2; Y3: year 3.(TIF)

S4 Fig Anti-PGL-I values (shown in units) in FSB samples of incident PB (n = 14) and MB (n = 7) leprosy patients at diagnosis. Open circles represent anti-PGL-I IgM units of individual samples. Units above dotted line (units ≥ 1) are considered seropositive. Solid black circles indicate BI-positive individuals. The Mann-Whitney U test was applied to assess statistical significance between the two groups. BI: bacterial index; MB: multibacillary; PB: paucibacillary.
(TIF)

S5 Fig Anti-PGL-I values (shown in units) of contacts on Anjouan, Mohéli and Madagascar testing seropositive in year 1 longitudinally. Anti-PGL-I IgM (*y*-axis) units of contacts on Anjouan (left panel), Mohéli (middle panel) and Madagascar (right panel) testing seropositive in year 1 per study year (*x*-axis). Open circles represent anti-PGL-I units of individual samples. Units above dotted line (units ≥ 1) are considered seropositive. Friedman tests were performed to assess statistical significance between anti-PGL-I levels at the beginning and end of the study (****P ≤ 0.0001). PGL-I: phenolic glycolipid-I; Y1: year 1; Y2: year 2; Y3: year 3.
(TIF)

S6 Fig Longitudinal anti-PGL-I values (shown in units) among contacts on Anjouan, Mohéli and Madagascar testing seropositive in year 1 separated by SDDR-PEP intake. In both groups (SDDR-PEP and no PEP), active case finding took place. A: Paired median anti-PGL-I IgM (*y*-axis) units of seropositive contacts (Y1) that received SDDR-PEP or not (*x*-axis) on Anjouan (left panel), Mohéli (middle panel) and Madagascar (right panel) longitudinally. Units above dotted line (units ≥ 1) are considered seropositive. Wilcoxon tests were performed to test for statistical significance between two groups (**P ≤ 0.01, ****P ≤ 0.0001). B: Paired percentages (%) of individuals testing seropositive for anti-PGL-I IgM (*y*-axis) per study year (*x*-axis) divided per study site and SDDR-PEP intake (upper panel: active case finding + SDDR-PEP; lower panel: active case finding). McNemar tests were performed to test for statistical significance between percentages (*P ≤ 0.05, ***P ≤ 0.001). IgM: immunoglobulin M; PGL-I: phenolic glycolipid-I; SDDR-PEP: single double-dose rifampicin post-exposure prophylaxis; Y1: year 1; Y3: year 3.
(TIF)

S1 Checklist Inclusivity in global research. (DOCX)
