# Supplementary material for: Large-scale field application of a fingerstick blood test for Mycobacterium leprae infection: Monitoring population-wide effects of case finding and post-exposure prophylaxis on transmission in the Comoros and Madagascar
Source: PLOS Glob Public Health. 2025 Dec 2;5(12):e0005270. doi: 10.1371/journal.pgph.0005270 (PMC12671774; doi:10.1371/journal.pgph.0005270)
Supplement: S1 Table — *No incident cases in Madagascar were tested for anti-PGL-I IgM. MB: multibacillary; PB: paucibacillary. (DOCX) [file pgph.0005270.s001.docx]

**S1 Table. Overview of contacts tested for anti-PGL-I prior to leprosy diagnosis.**

|  | |  |
| --- | --- | --- |
| Anjouan | total | 35 |
|  | sex |  |
|  | male | 20 (57.1%) |
|  | female | 15 (42.9%) |
|  | median age (range in years)  type of leprosy  MB  PB | 16 (3-78)  10 (28.6%)  25 (71.4%) |
| Mohéli | total | 2 |
|  | sex |  |
|  | male | 2 (100%) |
|  | female | 0 (0%) |
|  | median age (range in years)  type of leprosy  MB  PB | 22 (11-33)  2 (100%)  0 (0%) |
| all | total | 37* |

*No incident cases in Madagascar were tested for anti-PGL-I IgM.
MB: multibacillary; PB: paucibacillary.
