# Supplementary figures and images for: Large-scale field application of a fingerstick blood test for Mycobacterium leprae infection: Monitoring population-wide effects of case finding and post-exposure prophylaxis on transmission in the Comoros and Madagascar
Source: PLOS Glob Public Health. 2025 Dec 2;5(12):e0005270. doi: 10.1371/journal.pgph.0005270 (PMC12671774; doi:10.1371/journal.pgph.0005270)

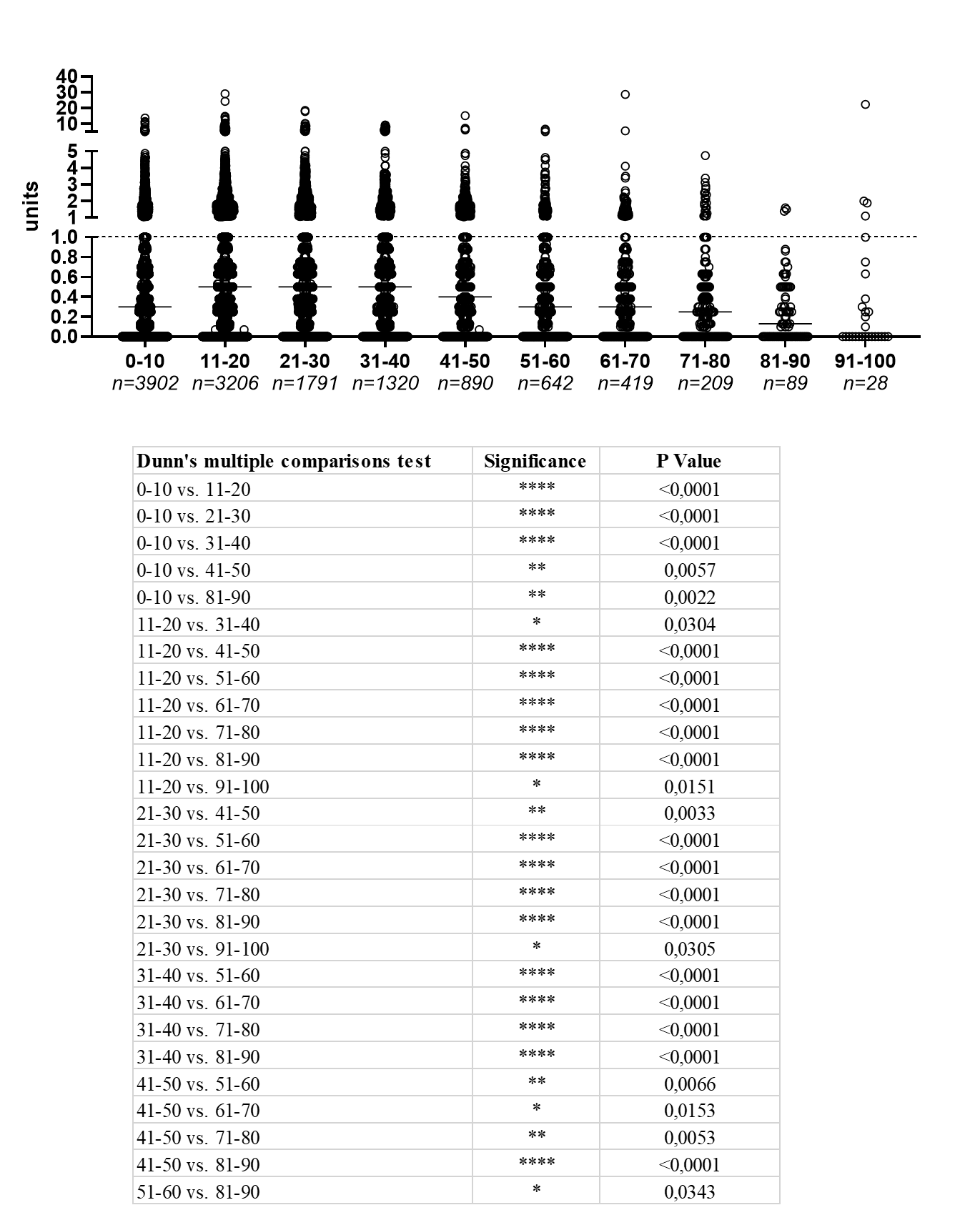

Supplement: S1 Fig — Open circles represent anti-PGL-I IgM units of individual samples. Units above dotted line (units ≥ 1) are considered seropositive. Kruskal-Wallis tests with Dunn’s correction for multiple testing were performed to assess statistical significance between anti-PGL-I units per age group. (TIF) [file pgph.0005270.s002.tif]

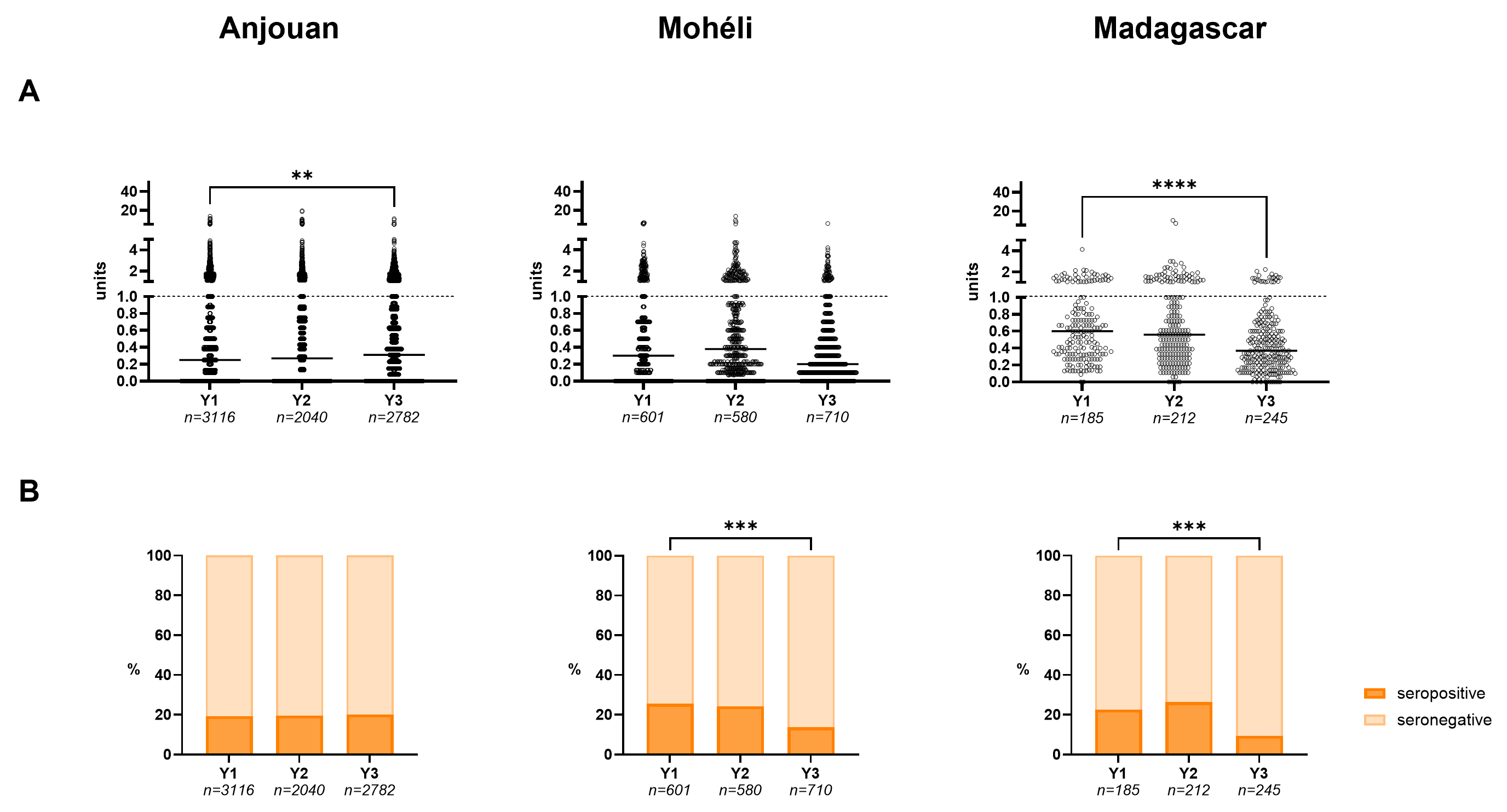

Supplement: S2 Fig — A: Anti-PGL-I IgM (y-axis) units of children (2–10 years of age) who are contacts of leprosy patients on Anjouan (left panel), Mohéli (middle panel) and Madagascar (right panel) per study year (x-axis). Open circles represent anti-PGL-I units of individual samples. Units above dotted line (units ≥ 1) are considered seropositive. Kruskal-Wallis tests were performed to assess statistical significance between anti-PGL-I units at the beginning and end of the study (**P ≤ 0.01, ****P ≤ 0.0001). B: Percentages (%) of child contacts (2–10 years of age) testing seropositive for anti-PGL-I IgM (y-axis) depicted per study site and year (x-axis). Chi-squared tests were performed to assess statistical significance between percentages at the beginning and end of the study (***P ≤ 0.001). FSB: fingerstick blood; IgM: immunoglobulin M; PGL-I: phenolic glycolipid-I; Y1: year 1; Y2: year 2; Y3: year 3. (TIF) [file pgph.0005270.s003.tif]

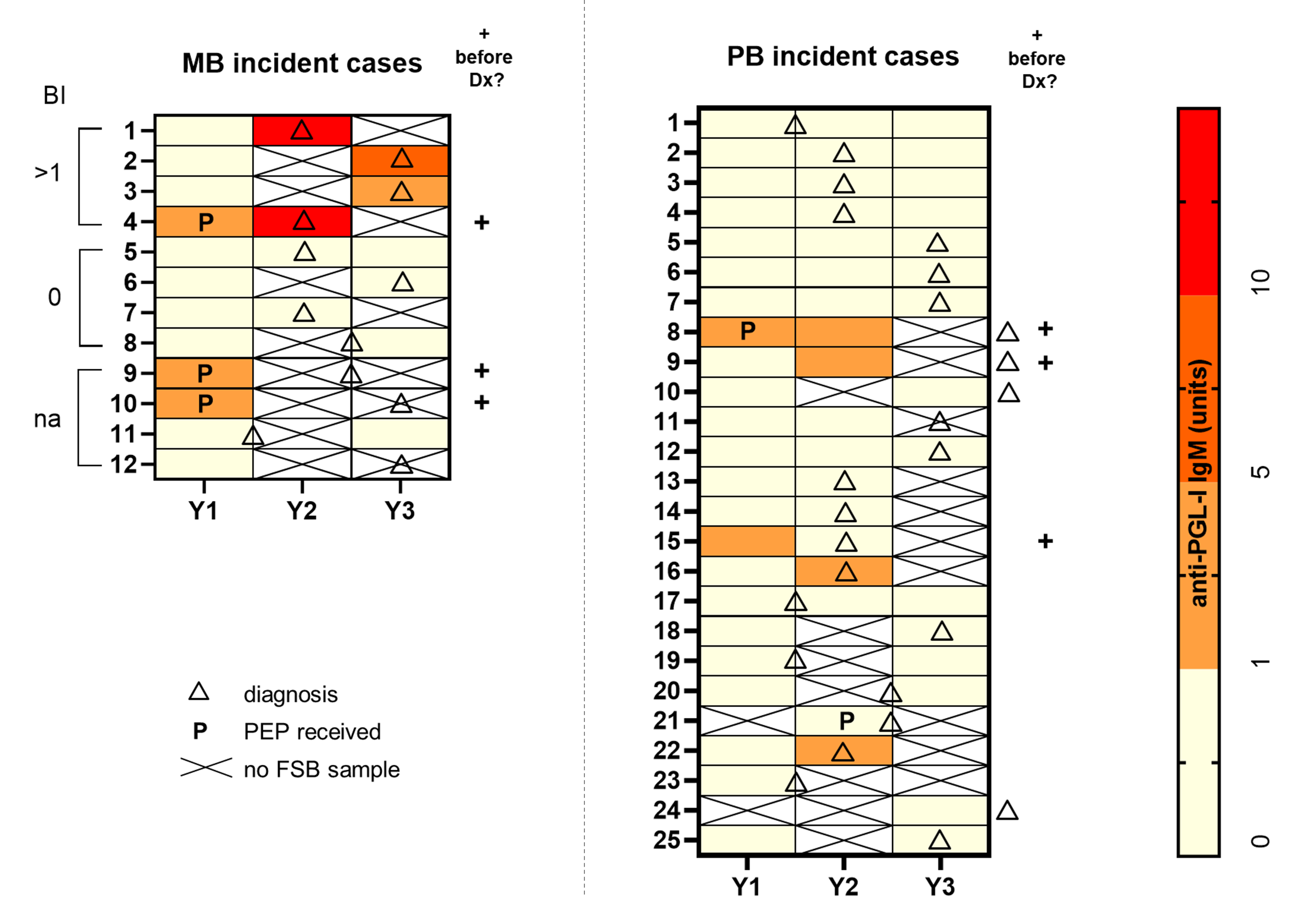

Supplement: S3 Fig — Heat maps showing anti-PGL-I IgM units of MB (left panel; n = 12) and PB (right panel; n = 25) leprosy cases. Anti-PGL-I units ≥ 1 are considered seropositive. ‘P’ indicates timepoint of receiving SDDR-PEP. Triangles indicate timepoint of diagnosis. ‘’ indicates that no FSB sample was taken in that year. Four contacts were diagnosed with leprosy after the third year of the study. Anti-PGL-I units 0-0.99: light orange; units 1-4.99: orange; units 5-9.99: dark orange; units ≥ 10: red. BI: bacterial index; FSB: fingerstick blood; MB: multibacillary; na: not available; PB: paucibacillary; PGL-I: phenolic glycolipid-I; SDDR-PEP: single double-dose rifampicin post exposure prophylaxis; Y1: year 1; Y2: year 2; Y3: year 3. (TIF) [file pgph.0005270.s004.tif]

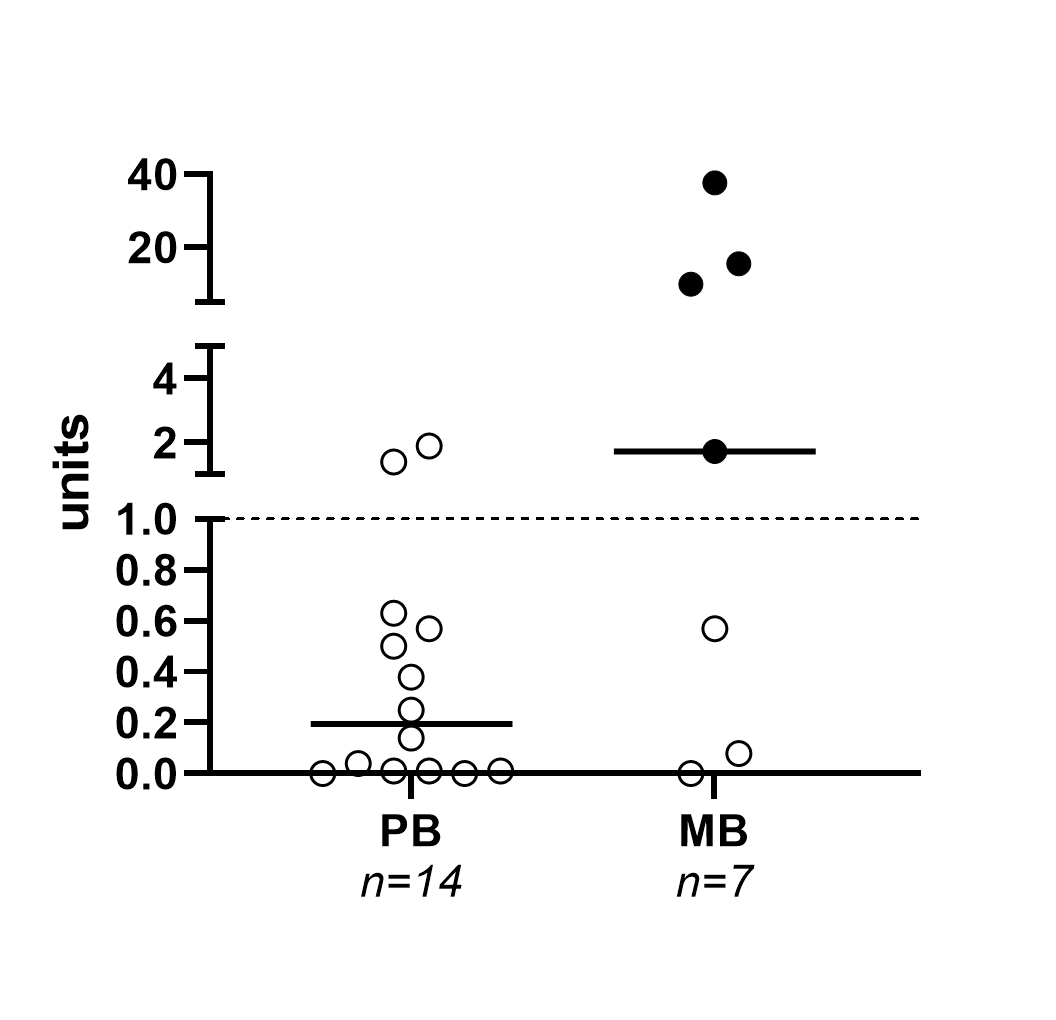

Supplement: S4 Fig — Open circles represent anti-PGL-I IgM units of individual samples. Units above dotted line (units ≥ 1) are considered seropositive. Solid black circles indicate BI-positive individuals. The Mann-Whitney U test was applied to assess statistical significance between the two groups. BI: bacterial index; MB: multibacillary; PB: paucibacillary. (TIF) [file pgph.0005270.s005.tif]

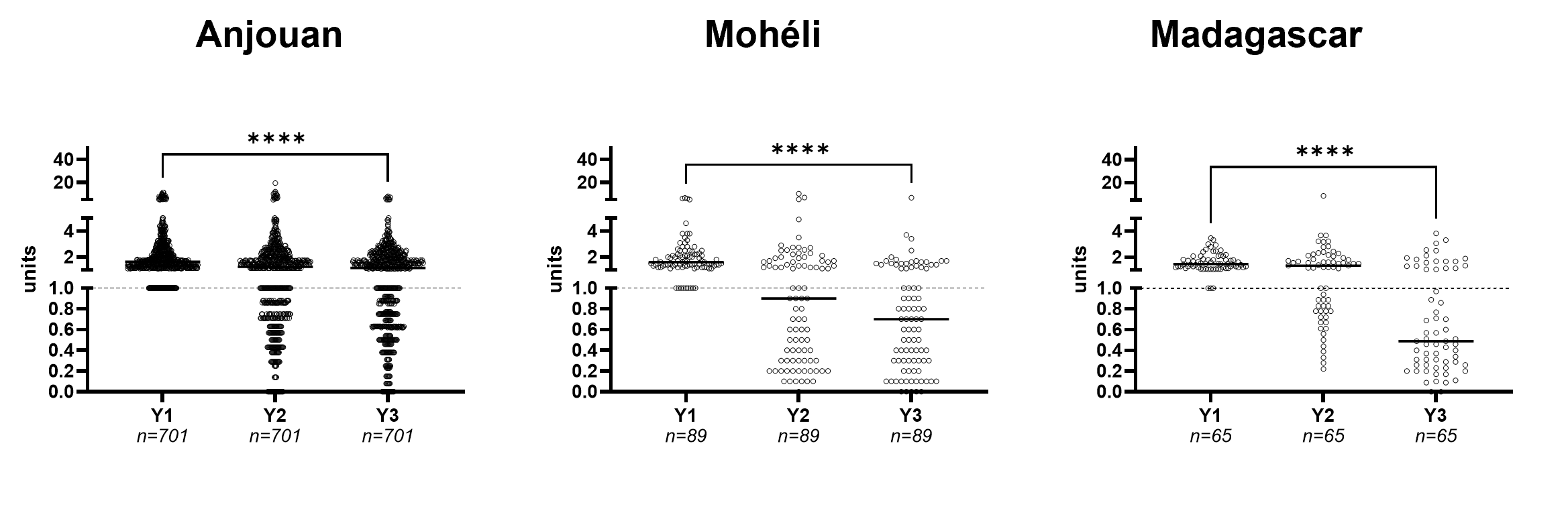

Supplement: S5 Fig — Anti-PGL-I IgM (y-axis) units of contacts on Anjouan (left panel), Mohéli (middle panel) and Madagascar (right panel) testing seropositive in year 1 per study year (x-axis). Open circles represent anti-PGL-I units of individual samples. Units above dotted line (units ≥ 1) are considered seropositive. Friedman tests were performed to assess statistical significance between anti-PGL-I levels at the beginning and end of the study (****P ≤ 0.0001). PGL-I: phenolic glycolipid-I; Y1: year 1; Y2: year 2; Y3: year 3. (TIF) [file pgph.0005270.s006.tif]

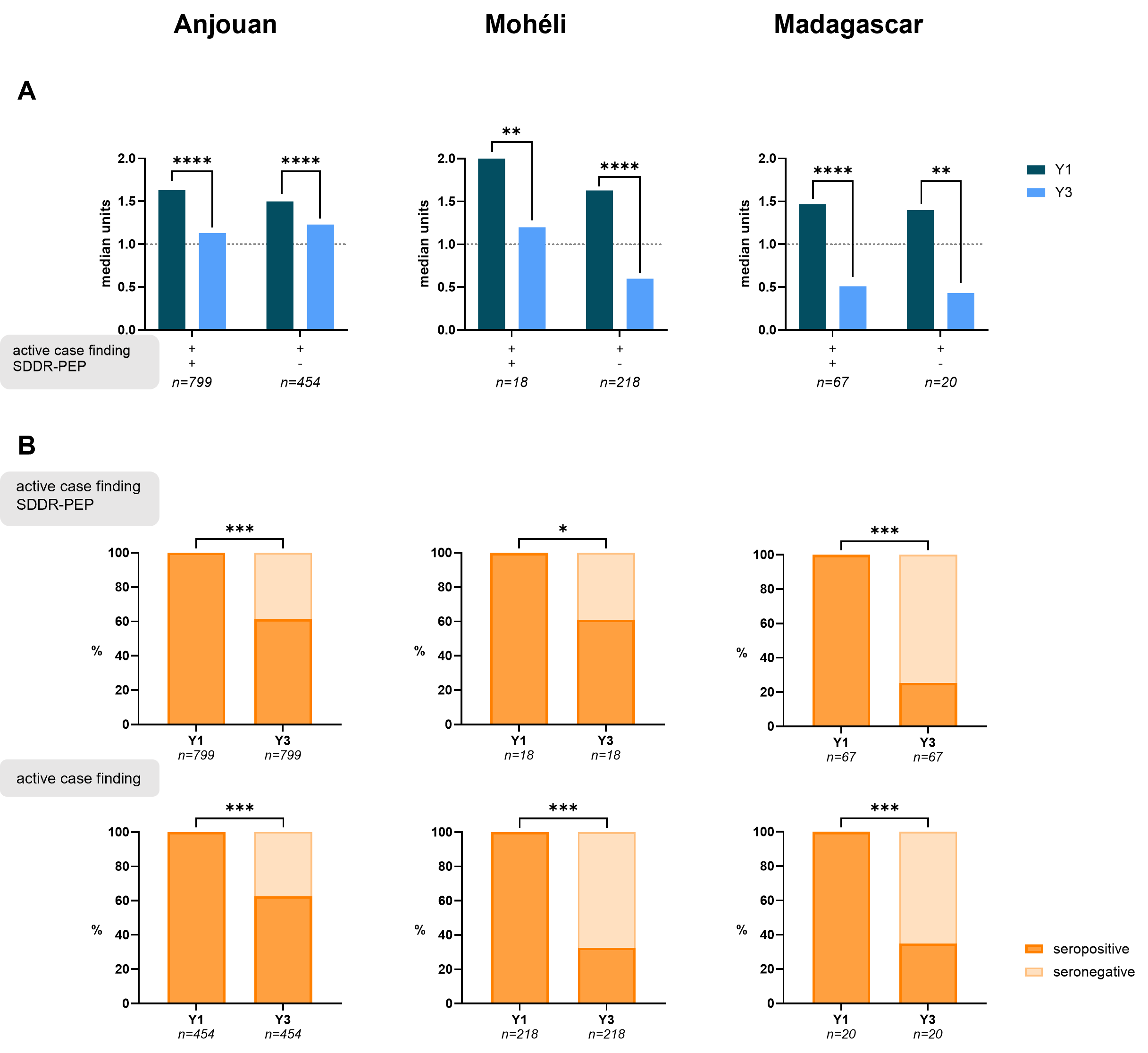

Supplement: S6 Fig — In both groups (SDDR-PEP and no PEP), active case finding took place. A: Paired median anti-PGL-I IgM (y-axis) units of seropositive contacts (Y1) that received SDDR-PEP or not (x-axis) on Anjouan (left panel), Mohéli (middle panel) and Madagascar (right panel) longitudinally. Units above dotted line (units ≥ 1) are considered seropositive. Wilcoxon tests were performed to test for statistical significance between two groups (**P ≤ 0.01, ****P ≤ 0.0001). B: Paired percentages (%) of individuals testing seropositive for anti-PGL-I IgM (y-axis) per study year (x-axis) divided per study site and SDDR-PEP intake (upper panel: active case finding + SDDR-PEP; lower panel: active case finding). McNemar tests were performed to test for statistical significance between percentages (*P ≤ 0.05, ***P ≤ 0.001). IgM: immunoglobulin M; PGL-I: phenolic glycolipid-I; SDDR-PEP: single double-dose rifampicin post-exposure prophylaxis; Y1: year 1; Y3: year 3. (TIF) [file pgph.0005270.s007.tif]
